# Relationship between blood manganese and bone mineral density and bone mineral content in adults: A population-based cross-sectional study

**DOI:** 10.1371/journal.pone.0276551

**Published:** 2022-10-21

**Authors:** Chao Wang, Yong Zhu, Haitao Long, Mingning Ou, Shushan Zhao

**Affiliations:** 1 Department of Orthopaedics, Xiangya Hospital, Central South University, Changsha, Hunan, China; 2 National Clinical Research Center for Geriatric Disorders, Xiangya Hospital, Central South University, Changsha, Hunan, China; The University of Mississippi Medical Center, UNITED STATES

## Abstract

**Purpose:**

It has been reported that bone is the primary organ for manganese (Mn) accumulation, but the association between manganese and bone loss remains debatable. Therefore, this study aimed to evaluate the relationship between blood manganese and bone mineral density/bone mineral content (BMD/BMC) by using a representative sample from the National Health and Nutrition Examination Survey (NHANES).

**Methods:**

A total of 9732 subjects over the age of 18 with available data were enrolled in this study. The relationship between blood manganese and BMD/BMC of the total body, spine and femoral regions was evaluated using multivariate linear regression models. Subgroup analyses were also performed.

**Results:**

We observed a negative association between blood manganese and BMD/BMC in the femoral neck and total body in the fully adjusted model, especially femoral neck BMD in women aged 50–70 years.

**Conclusion:**

In brief, people exposed to manganese should be aware of the increased risk of osteopenia or osteoporosis. Besides, due to the lack of available data, there are no definite values for the tolerable upper intake level (UL), average requirement (AR) and population reference intake (PRI) of manganese. The results of our study may provide some references for the establishment of AR, PRI and UL of Mn.

## Introduction

Osteoporosis is a disease characterized by low bone mass and destruction of bone structure, resulting in decreased bone strength and increased risk of fractures [1]. Osteoporotic fractures, also known as fragility fractures, are a major complication of osteoporosis, often occurring in the spine, hip, distal forearm, and proximal humerus. These fractures often lead to chronic pain, depression, disability, reduced life quality and increased mortality. The incidence of osteoporosis will rise as the longevity of global population significantly raised, with significant increases in morbidity, disability and mortality, resulting in a significant socio-economic burden [2]. The annual number of fragility fractures in the Europe is projected to increase from 3.5 million in 2010 to 4.5 million in 2025, with a significant loss of quality-adjusted life years (QALYs) and a significant increase in economic burden [1, 3]. For the diagnosis of osteoporosis, the T-score should be reported in postmenopausal women and in men aged 50 years or older. According to the WHO diagnostic criteria, osteoporosis is defined as a bone mineral density (BMD) T score of –2·5 or less. While for premenopausal women and men younger than 50 years, Z scores, not T-scores, are preferred, as provides a comparison of the patient’s BMD with average age-, sex-, and race-matched BMD and Z score ≤–2.0 is defined as “below the expected range for age” [4]. An estimated 16.2% of adults aged 65 and older in the United States had osteoporosis in the lumbar spine or femoral neck in 2010 [5]. Oral bisphosphonates remain the most cost-effective first-line treatment. Since there are sufficient effective treatments for osteoporosis, better methods are needed to identify patients with high risk of fracture [2].

Manganese (Mn), the trace element, is critical for many physiological and biological processes, including body growth, enzymatic regulation reactions, immune function, metabolism, and bone growth [6, 7]. However, excessive manganese in human body also causes harm to health. Manganese is partially absorbed through the gastrointestinal tract (3%-5%), vegetarians who eat foods rich in manganese, such as grains, legumes and nuts, and heavy tea drinkers, are likely having higher intake than the general population. People who smoke or inhale secondhand smoke are also exposed to higher levels of manganese than nonsmokers. Due to occupational exposure, the main source of entry for Mn is inhalation, which is also the primary source of clinically identified Mn intoxication [8]. Recently, due to methylcyclopentadienyl manganese tricarbonyl (MMT) used in gasoline [9] and contamination of drinking water [10, 11] more researchers have concerns over increasing manganese exposure in extensive environmental contamination.

Bone is the primary organ for Mn accumulation [12], but the association between Mn and bone loss remains debatable. Some previous studies have shown that women with osteoporosis have lower serum manganese levels than women with normal bone mineral density [13]. While a previous case-control study suggested that retired female workers in the highest manganese exposure model (tertile 3 of Mn-CEI) may be at higher risk of osteoporosis [14]. Besides, the European Food Safety Agency (EFSA) in 2013, judged that the available data to date are insufficient to establish an average requirement (Average Requirement, AR), as well as a reference intake for the population (Population Reference Intake, PRI) for Mn, and a tolerable upper intake level of manganese has not been established yet [15].

However, among these epidemiological studies, few studies have examined the relationship between manganese status and bone mineral density (BMD)/bone mineral content (BMC) in nationally representative samples. In this study, we used large datasets of total femur BMD/BMC, total spine BMD/BMC, and total body BMD/BMC released from 2013 to 2014 and 2017 to 2018 by the National Health and Nutrition Examination Survey (NHANES). We used blood manganese as a biomarker of manganese status. Therefore, the aim in this study was to evaluate the relationship between blood manganese and BMD/BMC by using a representative sample from the National Health and Nutrition Examination Survey. A better understanding of the relationship between blood manganese and BMD may help to better identify patients with osteoporosis and prevent osteoporotic fractures.

## Materials and methods

### Study design and population

**Fig 1:**
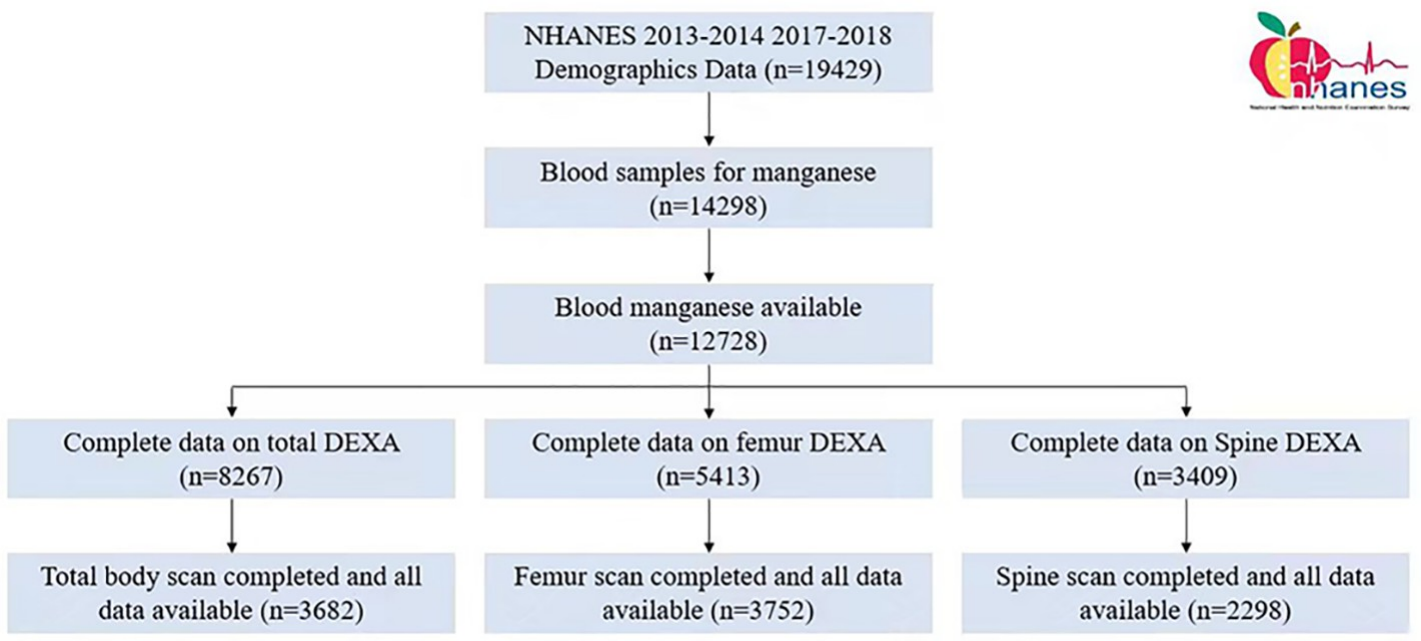
Flow chart. NHANES, National Health and Nutrition Examination Survey; DEXA, Dual Energy X-ray Absorptiometry.

The National Health and Nutrition Examination Survey (NHANES), a research program to assess the health and nutrition of adults and children in the United States, examining a nationally representative sample of approximately 5,000 people each year. In this study, we used NHANES data for 2013–2014 and 2017–2018. Of 19429 subjects, 12728 subjects with available blood manganese data were selected. After that, 2996 subjects lacking available Dual Energy X-ray Absorptiometry (DEXA) data were excluded. Finally, A total of 9732 subjects with available DEXA data on total body (n = 3682), femur (n = 3752), and spine (n = 2298) were enrolled. The Ethics Review Committee of the National Center for Health Statistics approved all NHANES protocols and obtained written informed consents from all participants [16]. The flow chart (Fig 1) also shows the selection process. All methods were performed in accordance with the relevant guidelines and regulations.

### Variables

The exposure variable was blood manganese, which was measured using mass spectrometry after a simple dilution sample preparation step. The Hologic Discovery A is a fan beam X-ray bone densitometer used to perform DXA examinations and estimate bone mineral content (BMC) and bone mineral density (BMD). DXA total-body scans will be performed on all individuals aged 18 to 59, while femur and spinal scans will be performed on samples aged 40 and older. Dependent variables were bone mineral density and bone mineral content of femur, lumbar spine and total body as measured by dual-energy X-ray absorptiometry. The following categorical variables were included as covariates into our analysis: gender, race/ethnicity (Mexican American, other Hispanic, non-Hispanic White, non-Hispanic Black, other races), drinking, hypertension, hypercholesterolemia, diabetes, difficulty walking or climbing stairs, renal insufficiency, asthma, arthritis, congestive heart failure, stroke, coronary heart disease, angina, thyroid problems, COPD, smoking, family smokers and osteoporosis. Our analysis included continuous covariates: age, body weight, standing height, and body mass index. Detailed information on blood manganese, BMD, BMC and covariates can be found on the NHANES website.

### Statistical analysis

Data were analyzed using R software (version 3.6.1), IBM SPSS Statistics version 26 (IBM, Armonk, New York, USA) and GraphPad Prism version 8.3.0 (GraphPad Software, San Diego, California USA). Means with standard deviation (SD) were used for continuous characteristic variables and categorical variables were expressed as percentages or frequencies. Differences in categorical variables between exposed groups were analyzed by Pearson’s chi-square tests. One-way ANOVA was used to analyze the differences of continuous variables among groups. Blood manganese levels were classified according to quartiles (quartile 1: < 25th percentile, quartile 2: 25th–50th percentile, quartile 3: 50th–75th percentile, quartile 4: > 75th percentile) and univariate and multivariable linear regression analysis were performed. In weighted univariate and multivariate linear regression models, we adjusted for potential confounders using an extended model approach to covariates to investigate the association between blood manganese levels and BMD/BMC (total femur, total spine, and total body). Including model 1 (unadjusted model), model 2 (adjusted for sex, age, race/ethnicity, and BMI), and model 3 (further adjusted for all relevant covariates in S1 Table). The results of regression analysis were represented by β regression coefficient, 95% confidence interval and P value, *P* < 0.05 (bilateral) was considered statistically significant.

## Results

### Participant characteristics

9732 subjects with available total body (n = 3682), femur (n = 3752), and spine (n = 2298) DEXA data were enrolled in this study. The average whole blood manganese level is 10.38 ± 3.85 ug/L (range: 1.88 to 52.0 ug/L) in total body DEXA data, 9.64 ± 3.67 ug/L (range: 1.57 to 54.92 ug/L) in femur DEXA data, 9.81 ± 3.47 ug/L (range: 1.57 to 35.56 ug/L) in spine DEXA data. Classified based on quartiles, the weighted characteristics of these participants are shown in S1 Table. In the femur DEXA data group, the weighted mean age of the participants was 62.1±10.7 years, 48.9% (n = 1838) were female, and there were significant differences in baseline characteristics among the groups in age, sex, race, body mass index, alcohol consumption, hypertension, osteoarthritis, stroke, thyroid problems, smoking, and family smoking (all *P* < 0.05). In the spine DEXA data group, the weighted mean age of participants was 59.6±10.8 years, 54.6% (n = 1254) were female, and there were significant differences in baseline characteristics among the groups in age, sex, race, body mass index, alcohol consumption, hypertension, osteoarthritis, stroke, smoking status, diabetes, and family smoking (all *P* < 0.05). In the total body DEXA data group, the weighted mean age of participants was 37.7±12.4 years and 50.2% (n = 1848) were female. There were significant differences in baseline characteristics among the groups in age, sex, race, body mass index, hypercholesterolemia, thyroid problems, smoking, and family smoking.

### Relationship between blood manganese levels and bone mineral density

Detailed results are represented in Tables 1–3 and S2 Table. Blood manganese levels are classified according to quartiles, the trends for blood manganese have statistical significance for left arm BMD (*P* for trend < 0.01); left leg BMD (*P* for trend < 0.01); right arm BMD (*P* for trend < 0.01); right leg BMD (*P* for trend < 0.01); left ribs BMD (*P* for trend < 0.01); thoracic spine BMD (*P* for trend < 0.05); lumber spine BMD (*P* for trend < 0.01); pelvis BMD (*P* for trend < 0.01); trunk bone BMD (*P* for trend < 0.01); subtotal BMD (*P* for trend < 0.01); total BMD (*P* for trend < 0.01) in total body.

**Table 1:** The relationship between blood manganese and BMD/BMC in femur.

		Model 1	Model 2	Model 3
		β (95%CI) *P* value	β (95%CI) *P* value	β (95%CI) *P* value
Total femur BMD(g/cm2)	Blood manganese(ug/L)	-0.0615(-0.0934,-0.0296) <0.001	-0.0174(-0.0429,0.0081) 0.183	-0.0224(-0.0506,0.0058) 0.122
	Q1:1.57–7.29 ug/L	Reference	Reference	Reference
	Q2:7.30–9.12 ug/L	-0.0151(-0.1055,0.0753) 0.743	0.0069(-0.0641,0.0779) 0.849	-0.005(-0.0816,0.0716) 0.898
	Q3:9.13–11.34 ug/L	-0.0514(-0.1418,0.039) 0.265	-0.0031(-0.0742,0.068) 0.932	-0.016(-0.0936,0.0616) 0.687
	Q4:11.35–54.92 ug/L	-0.155(-0.2454,-0.0646) <0.001	-0.0351(-0.1074,0.0372) 0.341	-0.0301(-0.1105,0.0503) 0.463
	*P* for trend	0.004		
Total femur BMC(g)	Blood manganese(ug/L)	-0.162(-0.1936,-0.1304) <0.001	-0.0528(-0.0738,-0.0318) <0.001	-0.052(-0.0755,-0.0285) <0.001
	Q1:1.57–7.29 ug/L	Reference	Reference	Reference
	Q2:7.30–9.12 ug/L	-0.1342(-0.2236,-0.0448) 0.003	-0.0435(-0.1017,0.0147) 0.14321	-0.0523(-0.1158,0.0112) 0.106
	Q3:9.13–11.34 ug/L	-0.2537(-0.3431,-0.1643) <0.001	-0.0878(-0.1462,-0.0294) 0.003	-0.088(-0.1525,-0.0235) 0.007
	Q4:11.35–54.92 ug/L	-0.454(-0.5434,-0.3646) <0.001	-0.1467(-0.2061,-0.0873) <0.001	-0.134(-0.2006,-0.0674) <0.001
	*P* for trend	<0.001		
Femoral neck BMD(g/cm2)	Blood manganese(ug/L)	-0.0526(-0.0845,-0.0207) 0.001	-0.0361(-0.0637,-0.0085) 0.01	-0.0393(-0.0699,-0.0087) 0.012
	Q1:1.57–7.29 ug/L	Reference	Reference	Reference
	Q2:7.30–9.12 ug/L	-0.0255(-0.1161,0.0651) 0.581	-0.0223(-0.0989,0.0543) 0.569	-0.0451(-0.128,0.0378) 0.286
	Q3:9.13–11.34 ug/L	-0.022(-0.1126,0.0686) 0.634	-0.0118(-0.0886,0.065) 0.763	-0.0167(-0.101,0.0676) 0.698
	Q4:11.35–54.92 ug/L	-0.1287(-0.2193,-0.0381) 0.005	-0.0847(-0.1629,-0.0065) 0.034	-0.0865(-0.1735,0.0005) 0.052
	*P* for trend	0.034		
Femoral neck BMC(g)	Blood manganese(ug/L)	-0.1241(-0.1559,-0.0923) <0.001	-0.0544(-0.0795,-0.0293) <0.001	-0.0564(-0.0844,-0.0284) <0.001
	Q1:1.57–7.29 ug/L	Reference	Reference	Reference
	Q2:7.30–9.12 ug/L	-0.0794(-0.1694,0.0106) 0.083	-0.0198(-0.0892,0.0496) 0.577	-0.0324(-0.1081,0.0433) 0.401
	Q3:9.13–11.34 ug/L	-0.1331(-0.2231,-0.0431) 0.004	-0.0278(-0.0974,0.0418) 0.434	-0.0243(-0.1011,0.0525) 0.535
	Q4:11.35–54.92 ug/L	-0.3397(-0.4297,-0.2497) <0.001	-0.142(-0.213,-0.071) <0.001	-0.1398(-0.2194,-0.0602) <0.001
	*P* for trend	<0.001		
Trochanter BMD(g/cm2)	Blood manganese(ug/L)	-0.0443(-0.0762,-0.0124) 0.007	-0.0056(-0.0326,0.0214) 0.685	-0.0126(-0.0426,0.0174) 0.41221
	Q1:1.57–7.29 ug/L	Reference	Reference	Reference
	Q2:7.30–9.12 ug/L	-0.0033(-0.0939,0.0873) 0.943	0.0143(-0.0606,0.0892) 0.707	0.0071(-0.074,0.0882) 0.864
	Q3:9.13–11.34 ug/L	-0.0265(-0.1171,0.0641) 0.566	0.0151(-0.06,0.0902) 0.693	-0.002(-0.0843,0.0803) 0.962
	Q4:11.35–54.92 ug/L	-0.1045(-0.1951,-0.0139) 0.024	0.0005(-0.0757,0.0767) 0.989	0.003(-0.0821,0.0881) 0.944
	*P* for trend	0.101		
Trochanter BMC(g)	Blood manganese(ug/L)	-0.1481(-0.1799,-0.1163) <0.001	-0.0487(-0.0726,-0.0248) <0.001	-0.0528(-0.0798,-0.0258) <0.001
	Q1:1.57–7.29 ug/L	Reference	Reference	Reference
	Q2:7.30–9.12 ug/L	-0.136(-0.2256,-0.0464) 0.003	-0.0509(-0.1171,0.0153) 0.133	-0.0611(-0.134,0.0118) 0.101
	Q3:9.13–11.34 ug/L	-0.2302(-0.3198,-0.1406) <0.001	-0.0756(-0.142,-0.0092) 0.026	-0.0927(-0.1668,-0.0186) 0.014
	Q4:11.35–54.92 ug/L	-0.4061(-0.4957,-0.3165) <0.001	-0.1254(-0.193,-0.0578) <0.001	-0.1209(-0.1975,-0.0443) 0.002
	*P* for trend	<0.001		
Intertrochanter BMD(g/cm2)	Blood manganese(ug/L)	-0.06(-0.0919,-0.0281) <0.001	-0.0156(-0.0417,0.0105) 0.239	-0.0217(-0.0505,0.0071) 0.14
	Q1:1.57–7.29 ug/L	Reference	Reference	Reference
	Q2:7.30–9.12 ug/L	-0.0138(-0.1042,0.0766) 0.764	0.0099(-0.0622,0.082) 0.787	-0.0023(-0.0803,0.0757) 0.953
	Q3:9.13–11.34 ug/L	-0.0528(-0.1432,0.0376) 0.253	-0.003(-0.0753,0.0693) 0.936	-0.0212(-0.1004,0.058) 0.599
	Q4:11.35–54.92 ug/L	-0.154(-0.2444,-0.0636) <0.001	-0.0331(-0.1068,0.0406) 0.379	-0.0297(-0.1116,0.0522) 0.477
	*P* for trend	0.004		
Intertrochanter BMC(g)	Blood manganese(ug/L)	-0.1614(-0.193,-0.1298) <0.001	-0.0505(-0.0715,-0.0295) <0.001	-0.0476(-0.0713,-0.0239) <0.001
	Q1:1.57–7.29 ug/L	Reference	Reference	Reference
	Q2:7.30–9.12 ug/L	-0.132(-0.2214,-0.0426) 0.004	-0.0411(-0.0995,0.0173) 0.168	-0.0483(-0.1122,0.0156) 0.139
	Q3:9.13–11.34 ug/L	-0.2618(-0.3512,-0.1724) <0.001	-0.0946(-0.1532,-0.036) 0.002	-0.089(-0.1539,-0.0241) 0.007
	Q4:11.35–54.92 ug/L	-0.4569(-0.5463,-0.3675) <0.001	-0.1454(-0.205,-0.0858) <0.001	-0.1291(-0.1961,-0.0621) <0.001
	*P* for trend	<0.001		
Wards triangle BMD(g/cm2)	Blood manganese(ug/L)	0.0061(-0.0258,0.038) 0.71	-0.0191(-0.0479,0.0097) 0.193	-0.0282(-0.0598,0.0034) 0.08
	Q1:1.57–7.29 ug/L	Reference	Reference	Reference
	Q2:7.30–9.12 ug/L	0.0337(-0.0569,0.1243) 0.466	0.0068(-0.073,0.0866) 0.868	-0.0153(-0.101,0.0704) 0.726
	Q3:9.13–11.34 ug/L	0.0954(0.0048,0.186) 0.039	0.0414(-0.0386,0.1214) 0.311	0.0211(-0.0657,0.1079) 0.634
	Q4:11.35–54.92 ug/L	0.0461(-0.0445,0.1367) 0.319	-0.0207(-0.102,0.0606) 0.618	-0.0265(-0.1163,0.0633) 0.564
	*P* for trend	0.246		
Wards triangle BMC(g)	Blood manganese(ug/L)	0.0119(-0.02,0.0438) 0.465	-0.0181(-0.0471,0.0109) 0.223	-0.0268(-0.0589,0.0053) 0.102
	Q1:1.57–7.29 ug/L	Reference	Reference	Reference
	Q2:7.30–9.12 ug/L	0.0213(-0.0693,0.1119) 0.645	-0.0092(-0.09,0.0716) 0.824	-0.0313(-0.1181,0.0555) 0.479
	Q3:9.13–11.34 ug/L	0.0897(-0.0009,0.1803) 0.052	0.0299(-0.051,0.1108) 0.469	0.0082(-0.0798,0.0962) 0.855
	Q4:11.35–54.92 ug/L	0.0589(-0.0317,0.1495) 0.202	-0.0215(-0.1038,0.0608) 0.609	-0.0231(-0.114,0.0678) 0.619
	*P* for trend	0.237		

Model 1: no covariates were adjusted. Model 2: age, sex, and race/ethnicity (Mexican American, other Hispanic, non-Hispanic White, non-Hispanic Black, other races), BMI were adjusted. Model 3: age, sex, race/ethnicity (Mexican American, other Hispanic, non-Hispanic White, non-Hispanic Black, other races), BMI, Drinking alcohol, Hypertension, Arthritis, Stroke, Thyroid problems, Smoking, Household smokers were adjusted.

**Table 2:** The relationship between blood manganese and BMD/BMC in spine.

		Model 1	Model 2	Model 3
		β (95%CI) *P* value	β (95%CI) *P* value	β (95%CI) *P* value
Total spine BMD(g/cm2)	Blood manganese(ug/L)	-0.0681(-0.1089,-0.0273) 0.001	-0.0354(-0.073,0.0022) 0.065	-0.0251(-0.0678,0.0176) 0.249
	Q1:1.57–7.27 ug/L	Reference	Reference	Reference
	Q2:7.28–9.03 ug/L	-0.0358(-0.1512,0.0796) 0.543	-0.0316(-0.1361,0.0729) 0.553	-0.0582(-0.1715,0.0551) 0.314
	Q3:9.04–11.18 ug/L	-0.0781(-0.1937,0.0375) 0.185	-0.0503(-0.1552,0.0546) 0.347	-0.0157(-0.1309,0.0995) 0.789
	Q4:11.19–35.56 ug/L	-0.205(-0.3201,-0.0899) <0.001	-0.1271(-0.2327,-0.0215) 0.018	-0.0829(-0.2007,0.0349) 0.168
	*P* for trend	0.003		
Total spine BMC(g)	Blood manganese(ug/L)	-0.1217(-0.1623,-0.0811) <0.001	-0.0541(-0.0894,-0.0188) 0.003	-0.0558(-0.0966,-0.015) 0.007
	Q1:1.57–7.27 ug/L	Reference	Reference	Reference
	Q2:7.28–9.03 ug/L	-0.1622(-0.2769,-0.0475) 0.006	-0.0867(-0.1847,0.0113) 0.083	-0.1071(-0.2153,0.0011) 0.053
	Q3:9.04–11.18 ug/L	-0.2378(-0.3527,-0.1229) <0.001	-0.1216(-0.22,-0.0232) 0.015	-0.0963(-0.2065,0.0139) 0.086
	Q4:11.19–35.56 ug/L	-0.3822(-0.4967,-0.2677) <0.001	-0.1946(-0.2938,-0.0954) <0.001	-0.1801(-0.2928,-0.0674) 0.002
	*P* for trend	<0.001		
L1 BMD(g/cm2)	Blood manganese(ug/L)	-0.0534(-0.0942,-0.0126) 0.011	-0.0208(-0.0578,0.0162) 0.271	-0.0068(-0.0487,0.0351) 0.751
	Q1:1.57–7.27 ug/L	Reference	Reference	Reference
	Q2:7.28–9.03 ug/L	-0.0077(-0.1231,0.1077) 0.896	-0.006(-0.1089,0.0969) 0.91	-0.0384(-0.1499,0.0731) 0.5
	Q3:9.04–11.18 ug/L	-0.0412(-0.1568,0.0744) 0.485	-0.016(-0.1193,0.0873) 0.762	0.0174(-0.0959,0.1307) 0.764
	Q4:11.19–35.56 ug/L	-0.163(-0.2782,-0.0478) 0.006	-0.0861(-0.1904,0.0182) 0.106	-0.0344(-0.1504,0.0816) 0.561
	*P* for trend	0.0212		
L1 BMC(g)	Blood manganese(ug/L)	-0.1175(-0.1581,-0.0769) <0.001	-0.0438(-0.0787,-0.0089) 0.014	-0.0407(-0.0813,-0.0001) 0.049
	Q1:1.57–7.27 ug/L	Reference	Reference	Reference
	Q2:7.28–9.03 ug/L	-0.1371(-0.252,-0.0222) 0.019	-0.0603(-0.1575,0.0369) 0.224	-0.088(-0.1956,0.0196) 0.109
	Q3:9.04–11.18 ug/L	-0.2168(-0.3319,-0.1017) <0.001	-0.0953(-0.1929,0.0023) 0.055	-0.0677(-0.1773,0.0419) 0.225
	Q4:11.19–35.56 ug/L	-0.354(-0.4685,-0.2395) <0.001	-0.1518(-0.2502,-0.0534) 0.003	-0.1279(-0.24,-0.0158) 0.025
	*P* for trend	<0.001		
L2 BMD(g/cm2)	Blood manganese(ug/L)	-0.0574(-0.0982,-0.0166) 0.006	-0.0264(-0.0638,0.011) 0.168	-0.0115(-0.054,0.031) 0.594
	Q1:1.57–7.27 ug/L	Reference	Reference	Reference
	Q2:7.28–9.03 ug/L	-0.0346(-0.15,0.0808) 0.557	-0.0309(-0.1352,0.0734) 0.561	-0.0541(-0.167,0.0588) 0.347
	Q3:9.04–11.18 ug/L	-0.0614(-0.177,0.0542) 0.298	-0.0364(-0.1411,0.0683) 0.495	0.0017(-0.1132,0.1166) 0.976
	Q4:11.19–35.56 ug/L	-0.1862(-0.3014,-0.071) 0.001	-0.1118(-0.2172,-0.0064) 0.038	-0.0543(-0.1717,0.0631) 0.364
	*P* for trend	0.0094		
L2 BMC(g)	Blood manganese(ug/L)	-0.1135(-0.1541,-0.0729) <0.001	-0.0433(-0.0784,-0.0082) 0.015	-0.0435(-0.0841,-0.0029) 0.035
	Q1:1.57–7.27 ug/L	Reference	Reference	Reference
	Q2:7.28–9.03 ug/L	-0.1428(-0.2575,-0.0281) 0.014	-0.0654(-0.1628,0.032) 0.188	-0.0881(-0.1957,0.0195) 0.109
	Q3:9.04–11.18 ug/L	-0.2051(-0.32,-0.0902) <0.001	-0.0875(-0.1853,0.0103) 0.079	-0.0554(-0.1648,0.054) 0.321
	Q4:11.19–35.56 ug/L	-0.3648(-0.4793,-0.2503) <0.001	-0.1705(-0.2691,-0.0719) <0.001	-0.1542(-0.2661,-0.0423) 0.007
	*P* for trend	<0.001		
L3 BMD(g/cm2)	Blood manganese(ug/L)	-0.0688(-0.1096,-0.028) <0.001	-0.0416(-0.08,-0.0032) 0.034	-0.0311(-0.075,0.0128) 0.165
	Q1:1.57–7.27 ug/L	Reference	Reference	Reference
	Q2:7.28–9.03 ug/L	-0.0215(-0.1369,0.0939) 0.715	-0.0194(-0.1262,0.0874) 0.722	-0.041(-0.1576,0.0756) 0.491
	Q3:9.04–11.18 ug/L	-0.0819(-0.1975,0.0337) 0.165	-0.0609(-0.1681,0.0463) 0.265	-0.0219(-0.1405,0.0967) 0.717
	Q4:11.19–35.56 ug/L	-0.1933(-0.3085,-0.0781) 0.001	-0.1294(-0.2376,-0.0212) 0.019	-0.0815(-0.203,0.04) 0.188
	*P* for trend	0.0042		
L3 BMC(g)	Blood manganese(ug/L)	-0.1226(-0.1632,-0.082) <0.001	-0.0591(-0.0952,-0.023) 0.001	-0.0602(-0.1019,-0.0185) 0.005
	Q1:1.57–7.27 ug/L	Reference	Reference	Reference
	Q2:7.28–9.03 ug/L	-0.1273(-0.242,-0.0126) 0.029	-0.0532(-0.1534,0.047) 0.297	-0.0715(-0.1822,0.0392) 0.206
	Q3:9.04–11.18 ug/L	-0.23(-0.3449,-0.1151) <0.001	-0.1192(-0.2197,-0.0187) 0.02	-0.1008(-0.2135,0.0119) 0.079
	Q4:11.19–35.56 ug/L	-0.3729(-0.4874,-0.2584) <0.001	-0.1959(-0.2972,-0.0946) <0.001	-0.1782(-0.2934,-0.063) 0.002
	*P* for trend	<0.001		
L4 BMD(g/cm2)	Blood manganese(ug/L)	-0.0778(-0.1186,-0.037) <0.001	-0.0391(-0.0771,-0.0011) 0.043	-0.0352(-0.0787,0.0083) 0.113
	Q1:1.57–7.27 ug/L	Reference	Reference	Reference
	Q2:7.28–9.03 ug/L	-0.0608(-0.1762,0.0546) 0.301	-0.0513(-0.1567,0.0541) 0.341	-0.0797(-0.1953,0.0359) 0.177
	Q3:9.04–11.18 ug/L	-0.0963(-0.2119,0.0193) 0.102	-0.0574(-0.1632,0.0484) 0.288	-0.0316(-0.1492,0.086) 0.599
	Q4:11.19–35.56 ug/L	-0.2186(-0.3337,-0.1035) <0.001	-0.1251(-0.2319,-0.0183) 0.022	-0.1003(-0.2206,0.02) 0.102
	*P* for trend	0.002		
L4 BMC(g)	Blood manganese(ug/L)	-0.1161(-0.1567,-0.0755) <0.001	-0.0478(-0.0837,-0.0119) 0.009	-0.0528(-0.0947,-0.0109) 0.014
	Q1:1.57–7.27 ug/L	Reference	Reference	Reference
	Q2:7.28–9.03 ug/L	-0.1937(-0.3084,-0.079) <0.001	-0.1243(-0.2241,-0.0245) 0.015	-0.1472(-0.2585,-0.0359) 0.009
	Q3:9.04–11.18 ug/L	-0.23(-0.3449,-0.1151) <0.001	-0.1155(-0.2157,-0.0153) 0.023	-0.0964(-0.2097,0.0169) 0.095
	Q4:11.19–35.56 ug/L	-0.3677(-0.4822,-0.2532) <0.001	-0.1812(-0.2821,-0.0803) <0.001	-0.179(-0.2948,-0.0632) 0.002
	*P* for trend	<0.001		

Model 1: no covariates were adjusted. Model 2: age, sex, and race/ethnicity (Mexican American, other Hispanic, non-Hispanic White, non-Hispanic Black, other races), BMI were adjusted. Model 3: age, sex, race/ethnicity (Mexican American, other Hispanic, non-Hispanic White, non-Hispanic Black, other races), BMI, Drinking alcohol, Hypertension, Arthritis, Stroke, Smoking status, Diabetes, Household smokers were adjusted.

**Table 3:** The relationship between blood manganese and BMD/BMC in total body.

		Model 1	Model 2	Model 3
		β (95%CI) *P* value	β (95%CI) *P* value	β (95%CI) *P* value
Head BMD(g/cm2)	Blood manganese(ug/L)	0.0165(-0.0158,0.0488) 0.318	-0.053(-0.0848,-0.0212) 0.001	-0.0593(-0.0969,-0.0217) 0.002
	Q1:1.88–7.76 ug/L	Reference	Reference	Reference
	Q2:7.77–9.69 ug/L	-0.1111(-0.2024,-0.0198) 0.017	-0.1525(-0.2391,-0.0659) <0.001	-0.1261(-0.2249,-0.0273) 0.012
	Q3:9.70–12.18 ug/L	-0.051(-0.1423,0.0403) 0.273	-0.1523(-0.2391,-0.0655) <0.001	-0.2033(-0.3025,-0.1041) <0.001
	Q4:12.19–52.0 ug/L	-0.0178(-0.1091,0.0735) 0.702	-0.1967(-0.2857,-0.1077) <0.001	-0.1984(-0.3017,-0.0951) <0.001
	*P* for trend	0.0799		
Head BMC(g)	Blood manganese(ug/L)	-0.0169(-0.0492,0.0154) 0.307	-0.0348(-0.0681,-0.0015) 0.041	-0.0468(-0.086,-0.0076) 0.019
	Q1:1.88–7.76 ug/L	Reference	Reference	Reference
	Q2:7.77–9.69 ug/L	-0.1346(-0.2259,-0.0433) 0.004	-0.1471(-0.2378,-0.0564) 0.001	-0.1109(-0.214,-0.0078) 0.035
	Q3:9.70–12.18 ug/L	-0.1119(-0.203,-0.0208) 0.016	-0.1336(-0.2247,-0.0425) 0.004	-0.1831(-0.2866,-0.0796) <0.001
	Q4:12.19–52.0 ug/L	-0.0993(-0.1906,-0.008) 0.033	-0.1488(-0.2419,-0.0557) 0.002	-0.1628(-0.2706,-0.055) 0.003
	*P* for trend	0.02		
Left Arm BMD(g/cm2)	Blood manganese(ug/L)	-0.2316(-0.263,-0.2002) <0.001	-0.0759(-0.0992,-0.0526) <0.001	-0.0665(-0.0941,-0.0389) <0.001
	Q1:1.88–7.76 ug/L	Reference	Reference	Reference
	Q2:7.77–9.69 ug/L	-0.1406(-0.2298,-0.0514) 0.002	-0.0737(-0.1374,-0.01) 0.023	-0.0162(-0.0887,0.0563) 0.662
	Q3:9.70–12.18 ug/L	-0.2937(-0.3827,-0.2047) <0.001	-0.1378(-0.2017,-0.0739) <0.001	-0.121(-0.1939,-0.0481) 0.001
	Q4:12.19–52.0 ug/L	-0.604(-0.693,-0.515) <0.001	-0.2169(-0.2822,-0.1516) <0.001	-0.1807(-0.2566,-0.1048) <0.001
	*P* for trend	<0.001		
Left Arm BMC(g)	Blood manganese(ug/L)	-0.257(-0.2882,-0.2258) <0.001	-0.1035(-0.1249,-0.0821) <0.001	-0.0948(-0.1203,-0.0693) <0.001
	Q1:1.88–7.76 ug/L	Reference	Reference	Reference
	Q2:7.77–9.69 ug/L	-0.1686(-0.257,-0.0802) <0.001	-0.1071(-0.1655,-0.0487) <0.001	-0.0755(-0.1423,-0.0087) 0.027
	Q3:9.70–12.18 ug/L	-0.3339(-0.4221,-0.2457) <0.001	-0.1923(-0.2509,-0.1337) <0.001	-0.1783(-0.2453,-0.1113) <0.001
	Q4:12.19–52.0 ug/L	-0.7031(-0.7913,-0.6149) <0.001	-0.3265(-0.3865,-0.2665) <0.001	-0.2963(-0.3661,-0.2265) <0.001
	*P* for trend	<0.001		
Left Leg BMD(g/cm2)	Blood manganese(ug/L)	-0.1881(-0.2199,-0.1563) <0.001	-0.0799(-0.1068,-0.053) <0.001	-0.0698(-0.1016,-0.038) <0.001
	Q1:1.88–7.76 ug/L	Reference	Reference	Reference
	Q2:7.77–9.69 ug/L	-0.1032(-0.1932,-0.0132) 0.025	-0.0608(-0.1343,0.0127) 0.105	-0.0088(-0.0923,0.0747) 0.837
	Q3:9.70–12.18 ug/L	-0.2345(-0.3245,-0.1445) <0.001	-0.1316(-0.2053,-0.0579) <0.001	-0.1304(-0.2141,-0.0467) 0.002
	Q4:12.19–52.0 ug/L	-0.4773(-0.5673,-0.3873) <0.001	-0.2073(-0.2828,-0.1318) <0.001	-0.1803(-0.2675,-0.0931) <0.001
	*P* for trend	<0.001		
Left Leg BMC(g)	Blood manganese(ug/L)	-0.2421(-0.2735,-0.2107) <0.001	-0.1068(-0.1307,-0.0829) <0.001	-0.1048(-0.133,-0.0766) <0.001
	Q1:1.88–7.76 ug/L	Reference	Reference	Reference
	Q2:7.77–9.69 ug/L	-0.1491(-0.2379,-0.0603) 0.001	-0.094(-0.1591,-0.0289) 0.004	-0.0531(-0.1272,0.021) 0.16
	Q3:9.70–12.18 ug/L	-0.2977(-0.3863,-0.2091) <0.001	-0.1661(-0.2314,-0.1008) <0.001	-0.1562(-0.2305,-0.0819) <0.001
	Q4:12.19–52.0 ug/L	-0.6524(-0.741,-0.5638) <0.001	-0.318(-0.3846,-0.2514) <0.001	-0.3042(-0.3816,-0.2268) <0.001
	*P* for trend	<0.001		
Pelvis BMD(g/cm2)	Blood manganese(ug/L)	-0.0816(-0.1137,-0.0495) <0.001	-0.0515(-0.0834,-0.0196) 0.001	-0.0439(-0.0817,-0.0061) 0.023
	Q1:1.88–7.76 ug/L	Reference	Reference	Reference
	Q2:7.77–9.69 ug/L	-0.0608(-0.1519,0.0303) 0.191	-0.0601(-0.1475,0.0273) 0.178	0.0046(-0.0952,0.1044) 0.927
	Q3:9.70–12.18 ug/L	-0.0686(-0.1595,0.0223) 0.139	-0.0619(-0.1495,0.0257) 0.166	-0.0662(-0.1664,0.034) 0.195
	Q4:12.19–52.0 ug/L	-0.2266(-0.3177,-0.1355) <0.001	-0.1503(-0.2399,-0.0607) 0.001	-0.1366(-0.2409,-0.0323) 0.01
	*P* for trend	<0.001		
Pelvis BMC(g)	Blood manganese(ug/L)	-0.1768(-0.2086,-0.145) <0.001	-0.1042(-0.1334,-0.075) <0.001	-0.1083(-0.1424,-0.0742) <0.001
	Q1:1.88–7.76 ug/L	Reference	Reference	Reference
	Q2:7.77–9.69 ug/L	-0.135(-0.225,-0.045) 0.003	-0.1133(-0.1929,-0.0337) 0.005	-0.0639(-0.1539,0.0261) 0.164
	Q3:9.70–12.18 ug/L	-0.2222(-0.312,-0.1324) <0.001	-0.1462(-0.2262,-0.0662) <0.001	-0.136(-0.2264,-0.0456) 0.003
	Q4:12.19–52.0 ug/L	-0.5003(-0.5901,-0.4105) <0.001	-0.3168(-0.3985,-0.2351) <0.001	-0.3146(-0.4085,-0.2207) <0.001
	*P* for trend	<0.001		
Trunk Bone BMD(g/cm2)	Blood manganese(ug/L)	-0.1324(-0.1643,-0.1005) <0.001	-0.0872(-0.1186,-0.0558) <0.001	-0.0859(-0.1231,-0.0487) <0.001
	Q1:1.88–7.76 ug/L	Reference	Reference	Reference
	Q2:7.77–9.69 ug/L	-0.1022(-0.1929,-0.0115) 0.027	-0.0919(-0.1779,-0.0059) 0.036	-0.0383(-0.1367,0.0601) 0.444
	Q3:9.70–12.18 ug/L	-0.1748(-0.2654,-0.0842) <0.001	-0.1372(-0.2234,-0.051) 0.002	-0.1458(-0.2446,-0.047) 0.004
	Q4:12.19–52.0 ug/L	-0.3645(-0.4551,-0.2739) <0.001	-0.2504(-0.3386,-0.1622) <0.001	-0.2393(-0.342,-0.1366) <0.001
	*P* for trend	<0.001		
Trunk Bone BMC(g)	Blood manganese(ug/L)	-0.1706(-0.2024,-0.1388) <0.001	-0.0819(-0.1093,-0.0545) <0.001	-0.0852(-0.1175,-0.0529) <0.001
	Q1:1.88–7.76 ug/L	Reference	Reference	Reference
	Q2:7.77–9.69 ug/L	-0.1012(-0.1912,-0.0112) 0.028	-0.0777(-0.1524,-0.003) 0.042	-0.0329(-0.1184,0.0526) 0.449
	Q3:9.70–12.18 ug/L	-0.1884(-0.2784,-0.0984) <0.001	-0.1221(-0.1972,-0.047) 0.001	-0.1217(-0.2074,-0.036) 0.005
	Q4:12.19–52.0 ug/L	-0.4775(-0.5675,-0.3875) <0.001	-0.2583(-0.3349,-0.1817) <0.001	-0.2484(-0.3376,-0.1592) <0.001
	*P* for trend	<0.001		
Total body BMD(g/cm2)	Blood manganese(ug/L)	-0.1409(-0.1728,-0.109) <0.001	-0.0823(-0.1135,-0.0511) <0.001	-0.0789(-0.1157,-0.0421) <0.001
	Q1:1.88–7.76 ug/L	Reference	Reference	Reference
	Q2:7.77–9.69 ug/L	-0.1365(-0.2271,-0.0459) 0.003	-0.1168(-0.2019,-0.0317) 0.007	-0.0523(-0.1493,0.0447) 0.29
	Q3:9.70–12.18 ug/L	-0.2172(-0.3078,-0.1266) <0.001	-0.1641(-0.2494,-0.0788) <0.001	-0.1826(-0.28,-0.0852) <0.001
	Q4:12.19–52.0 ug/L	-0.3847(-0.4753,-0.2941) <0.001	-0.2389(-0.3261,-0.1517) <0.001	-0.2188(-0.3201,-0.1175) <0.001
	*P* for trend	<0.001		
Total body BMC(g)	Blood manganese(ug/L)	-0.217(-0.2486,-0.1854) <0.001	-0.1022(-0.1279,-0.0765) <0.001	-0.1025(-0.1329,-0.0721) <0.001
	Q1:1.88–7.76 ug/L	Reference	Reference	Reference
	Q2:7.77–9.69 ug/L	-0.1606(-0.2498,-0.0714) <0.001	-0.1189(-0.1889,-0.0489) <0.001	-0.0717(-0.1515,0.0081) 0.078
	Q3:9.70–12.18 ug/L	-0.2823(-0.3715,-0.1931) <0.001	-0.1796(-0.2498,-0.1094) <0.001	-0.1784(-0.2586,-0.0982) <0.001
	Q4:12.19–52.0 ug/L	-0.6033(-0.6925,-0.5141) <0.001	-0.3211(-0.3928,-0.2494) <0.001	-0.3068(-0.3903,-0.2233) <0.001
	*P* for trend	<0.001		

Model 1: no covariates were adjusted. Model 2: age, sex, and race/ethnicity (Mexican American, other Hispanic, non-Hispanic White, non-Hispanic Black, other races), BMI were adjusted. Model 3: age, sex, race/ethnicity (Mexican American, other Hispanic, non-Hispanic White, non-Hispanic Black, other races), BMI, Hypercholesterolemia, Thyroid problems, Smoking, Household smokers were adjusted.

In femur area the trends for blood manganese are statistically significant for total femur BMD (*P* for trend < 0.01); femoral neck BMD (*P* for trend < 0.05); intertrochanter BMD (*P* for trend < 0.01) and in lumbar spine area, the trends for blood manganese have statistical significance for total spine BMD (*P* for trend < 0.01); L1 BMD (*P* for trend < 0.05); L2 BMD (P for trend < 0.01); L3 BMD (*P* for trend < 0.01); L4 BMD (*P* for trend < 0.01).

Weighted univariate and multiple linear regression models are constructed in Tables 1–3. In the unadjusted model (Model 1), there is a significant negative correlation between increased blood manganese concentration and BMD of femur, spine and total body region [*P*<0.05 except for wards triangle BMD (β coefficient 0.0061; 95%CI -0.0258,0.038) and head BMD (β coefficient 0.0165; 95%CI -0.0158,0.0488)]. After adjusting for all the relevant covariables simultaneously(Model 3), femoral neck BMD(β coefficient -0.0393; 95%CI -0.0699,-0.0087); head BMD(β coefficient -0.0593; 95%CI -0.0969,-0.0217); left arm BMD(β coefficient -0.0665; 95%CI -0.0941,-0.0389); left leg BMD(β coefficient -0.0698; 95%CI -0.1016,-0.038); pelvis BMD(β coefficient -0.0439; 95%CI -0.0817,-0.0061); trunk bone BMD(β coefficient -0.0859; 95%CI -0.1231,-0.0487); Total BMD(β coefficient -0.0789; 95%CI -0.1157,-0.0421), decrease with increasing manganese levels, but not the spinal areas.

### Relationship between blood manganese levels and bone mineral content

Detailed results are represented in Tables 1–3 and S2 Table. Blood manganese levels are classified according to quartiles, the trends for blood manganese have statistical significance for all the BMC of femur, spine and total body region (all *P* for trend < 0.05). Weighted univariate and multiple linear regression models are constructed in Tables 1–3. In model 1, there was a significant negative correlation between increased blood manganese concentration and BMC of femur, spine and total body region [*P*<0.05 except for wards triangle BMC (β coefficient 0.0119; 95%CI -0.02, 0.0438) and head BMC (β coefficient -0.01685; 95%CI -0.0492, 0.0154)]. In model 3, BMC of femur, spine and total body, except wards triangle BMC (β coefficient -0.0268; 95%CI -0.0589, 0.0053; *P* 0.102), all decrease with increasing manganese levels.

### Subgroup analysis

Subgroup analyses after adjusted for all relevant covariates are shown in Tables 4–6. Multiple regression analyses in the femoral region have showed that, stratified by sex, femoral neck BMD decreases with increased blood manganese (Femoral neck BMD: β coefficient -0.0544; 95%CI -0.0989, -0.0099; *P* 0.017), and femoral neck BMC decreases with increased blood manganese in all gender groups (β coefficient < 0, *P* < 0.05). Results stratified by race have showed that there are no significant correlation between femoral neck BMD/BMC and blood manganese in all races. The results of stratification by age have showed that the BMD of femoral neck decreased with the increase of blood manganese in 50–59 and 60–69 years [(β coefficient -0.0581; 95%CI -0.1151, -0.0011; *P* 0.046); (β coefficient -0.0561. 95% CI -0.1104, -0.0018; *P* 0.043)], and there is a negative correlation between BMC and blood manganese.

**Table 4:** Association of blood manganese with femoral neck BMD/BMC, stratified by sex, race/ethnicity and age.

	Femoral neck BMD(g/cm^2^)		Femoral neck BMC(g)	
	β (95% CI)	*P* value	β (95% CI)	*P* value
Subgroup analysis stratified by sex				
Men	-0.0351(-0.0788,0.0086)	0.115	-0.0655(-0.1098,-0.0212)	0.004
Women	-0.0544(-0.0989,-0.0099)	0.017	-0.073(-0.1202,-0.0258)	0.003
Subgroup analysis stratified by race/ethnicity				
Mexican American	-0.0229(-0.1162,0.0704)	0.631	-0.0417(-0.1283,0.0449)	0.347
Other Hispanic	-0.0446(-0.1359,0.0467)	0.339	-0.0397(-0.1193,0.0399)	0.329
Non-Hispanic White	0.0114(-0.0354,0.0582)	0.634	-0.0102(-0.0525,0.0321)	0.637
Non-Hispanic Black	0.0349(-0.0312,0.101)	0.301	0.0321(-0.0281,0.0923)	0.296
Other Race—Including Multi-Racial	-0.0391(-0.2277,0.1495)	0.685	-0.0394(-0.2176,0.1388)	0.666
Subgroup analysis stratified by age				
40–49	0.0534(-0.0617,0.1685)	0.364	0.0244(-0.0783,0.1271)	0.642
50–59	-0.0581(-0.1151,-0.0011)	0.046	-0.0643(-0.1155,-0.0131)	0.014
60–69	-0.0561(-0.1104,-0.0018)	0.043	-0.0674(-0.1154,-0.0194)	0.006
70–80	-0.0094(-0.0706,0.0518)	0.764	-0.0448(-0.0989,0.0093)	0.105

Age, sex, race/ethnicity (Mexican American, other Hispanic, non-Hispanic White, non-Hispanic Black, other races), BMI, Drinking alcohol, Hypertension, Arthritis, Stroke, Thyroid problems, Smoking, Household smokers were adjusted. In the subgroup analysis stratified by sex or race/ethnicity, the model is not adjusted for the stratification variable itself.

**Table 5:** Association of blood manganese with total spine BMD/BMC, stratified by sex, race/ethnicity and age.

	Total spine BMD(g/cm^2^)		Total spine BMC(g)	
	β (95% CI)	*P* value	β (95% CI)	*P* value
Subgroup analysis stratified by sex				
Men	-0.043(-0.1045,0.0185)	0.171	-0.075(-0.1393,-0.0107)	0.022
Women	-0.0186(-0.0774,0.0402)	0.536	-0.057(-0.1211,0.0071)	0.082
Subgroup analysis stratified by race/ethnicity				
Mexican American	0.0591(-0.0593,0.1775)	0.328	0.0139(-0.0945,0.1223)	0.802
Other Hispanic	0.0141(-0.1035,0.1317)	0.814	0.0446(-0.0665,0.1557)	0.433
Non-Hispanic White	0.0005(-0.0685,0.0695)	0.988	-0.0366(-0.1015,0.0283)	0.269
Non-Hispanic Black	0.0653(-0.029,0.1596)	0.175	0.0348(-0.0552,0.1248)	0.449
Other Race—Including Multi-Racial	-0.0827(-0.381,0.2156)	0.59	-0.0239(-0.3273,0.2795)	0.878
Subgroup analysis stratified by age				
40–49	0.1017(-0.023,0.2264)	0.111	0.0204(-0.1011,0.1419)	0.742
50–59	-0.0978(-0.1697,-0.0259)	0.008	-0.1021(-0.1717,-0.0325)	0.004
60–69	0.0095(-0.0626,0.0816)	0.796	-0.0375(-0.1069,0.0319)	0.289
70–80	-0.0626(-0.1532,0.028)	0.176	-0.0653(-0.151,0.0204)	0.136

Age, sex, race/ethnicity (Mexican American, other Hispanic, non-Hispanic White, non-Hispanic Black, other races), BMI, Drinking alcohol, Hypertension, Arthritis, Stroke, Smoking status, Diabetes, Household smokers were adjusted. In the subgroup analysis stratified by sex or race/ethnicity, the model is not adjusted for the stratification variable itself.

**Table 6:** Association of blood manganese with total body BMD/BMC, stratified by sex, race/ethnicity and age.

	Total body BMD(g/cm^2^)		Total body BMC(g)	
	β (95% CI)	*P* value	β (95% CI)	*P* value
Subgroup analysis stratified by sex				
Men	-0.1455(-0.1976,-0.0934)	<0.001	-0.1861(-0.2355,-0.1367)	<0.001
Women	-0.0324(-0.0861,0.0213)	0.237	-0.0674(-0.118,-0.0168)	0.009
Subgroup analysis stratified by race/ethnicity				
Mexican American	-0.0455(-0.1347,0.0437)	0.319	-0.1157(-0.1855,-0.0459)	0.001
Other Hispanic	0.0139(-0.0988,0.1266)	0.808	0.0054(-0.0848,0.0956)	0.906
Non-Hispanic White	-0.0106(-0.0731,0.0519)	0.74	-0.0206(-0.0712,0.03)	0.426
Non-Hispanic Black	0.0033(-0.0863,0.0929)	0.943	0.0366(-0.0389,0.1121)	0.343
Other Race—Including Multi-Racial	-0.1046(-0.2596,0.0504)	0.188	-0.073(-0.1988,0.0528)	0.258
Subgroup analysis stratified by age				
18–29	-0.1255(-0.1976,-0.0534)	<0.001	-0.1402(-0.199,-0.0814)	<0.001
30–39	-0.0883(-0.1614,-0.0152)	0.018	-0.0769(-0.1367,-0.0171)	0.012
40–49	-0.0361(-0.1186,0.0464)	0.391	-0.1082(-0.1768,-0.0396)	0.002
50–59	-0.0688(-0.136,-0.0016)	0.046	-0.0934(-0.1493,-0.0375)	0.001

Age, sex, race/ethnicity (Mexican American, other Hispanic, non-Hispanic White, non-Hispanic Black, other races), BMI, Hypercholesterolemia, Thyroid problems, Smoking, Household smokers were adjusted. In the subgroup analysis stratified by sex or race/ethnicity, the model is not adjusted for the stratification variable itself.

In the spinal region, gender stratified results have showed that only the total spinal BMC is significantly correlated with the blood manganese, and no significant correlations are found between the total spinal BMD and the blood manganese in all gender groups. Results stratified by race have showed that there is no significant correlation between BMD/BMC and blood manganese in all races. After stratified by age, BMD/BMC is negatively correlated with blood manganese in the total spine at 50–59 years of age [(β coefficient -0.0978; 95%CI -0.1697, -0.0259; *P* 0.008); (β coefficient -0.1021; 95% CI -0.1717, -0.0325; *P* 0.004)].

In the total body region, after stratified by sex, BMD decreased significantly with blood manganese increasing in males (β coefficient -0.1455; 95% CI -0.1976, -0.0934; *P* <0.001), while total body BMC decreased with blood manganese increasing in all gender groups (β coefficient < 0, *P* <0.05). Results stratified by race have showed that there is no significant correlation between BMD/BMC of the total body and blood manganese in all races, except BMC in Mexican American (β coefficient -0.1157; 95% CI -0.1855, -0.0459; *P* 0.001). Results stratified by age have showed a significant negative correlation in most age groups between 18 to 59 (β coefficient < 0, *P* <0.05), except 40–49.

## Discussion

The incidence of osteoporosis is rising, with the increasing longevity of global population. According to International Commission on Radiation Protection (ICRP), about 40% of body manganese accumulates in bones [17]. Thus, we hypothesized that blood manganese might somehow be associated with BMD/BMC. In contrast to previous studies, after adjusting for confounders, we found that BMD in the femoral neck and total body is negatively associated with blood manganese, while BMD in the spine is not significantly negatively associated with blood manganese. The BMC of femur, spine and total body has showed significant negative correlation with blood manganese. Subgroup analysis have showed that for BMD, blood manganese is negatively correlated with femur neck BMD in females aged between 50–70 years, spinal BMD in 50–60 years old, and total body BMD in males and most ages of 18–60 years old. For BMC, blood manganese is inversely associated with femur neck BMC in males or females, 50–70 years of age, spinal BMC in males, 50–60 years of age, and total body BMC in males or females, Mexican Americans, and 18–60 years of age.

Multiple linear regression analysis and subgroup analysis have showed that the relationship between blood manganese and BMD has mainly been found on the femoral neck and total body, but not the spine. This may be attributed to the different structure of the femur and spine, as previous studies shown, the trabecular bone of the lumbar spine is dominant compared to the femoral neck, which contains a higher proportion of cortical bone. Although trabecular bone makes up only 20% of bone mass in healthy adult bones, it has a greater surface area and remodeling rate than cortical bone [18, 19]. However, due to the lack of research on the specific mechanism of blood manganese in bone metabolism, further basic research is necessary. Interestingly, it is noteworthy that no matter in femoral neck, spine or total body, blood manganese concentration is significantly negatively correlated with BMD/BMC in people between 50–60 years old, indicating that blood manganese concentration has certain predictive value for bone mineral density as well as bone mass in those between 50–60 years old. The explanation for this phenomenon is unclear, however, owing to changes in physical function, age may be a common factor influencing an individual’s susceptibility to manganese toxicity. In addition, our study has found that in the femoral neck, blood manganese is significantly negatively correlated with BMD in female instead of male, while in the total body, blood manganese is significantly negatively correlated with BMD in male rather than female. On the one way, it may account for this difference that the femur DEXA data focused on people over 40 years of age while the DEXA data on total body are concentrated in people aged 8–59, and the data under 18 years old are excluded in the study. On the other way, we speculate that different sex hormones levels may have different function on the BMD in femoral neck or total body.

Previous studies have shown that most changes in BMD, especially in the hip, can be attributed to BMC variation, and both are considered to be important determinants of osteoporotic fractures [20]. In our study, blood manganese is significantly negatively correlated with BMC in most regions of femur, spine and total body. It is consistent with previous finding that bone weight reduction was observed in rats with long-term oral manganese intake [21]. However, the association between blood manganese level and bone loss remained debatable, and some previous epidemiological studies have shown mixed findings. A study determining the effect of manganese (Mn) supplementation on BMD and bone metabolic parameters in ovariectomized Sprague-Dawley rats found that manganese supplementation improved mineral density and serum osteocalcin in the spine and femur of rats [22]. Another prospective cross-sectional study of 41 untreated postmenopausal women showed a significant positive association between serum manganese levels and lumbar or femoral BMD [23]. Odabasi et al., in a case-control study of 138 postmenopausal women, reported that there were no significant differences in blood manganese levels between postmenopausal women with osteoporosis and postmenopausal women without osteoporosis. The median blood manganese concentration was 14.76 ng/mL in the osteoporosis group and 15.54 ng/mL in the control group [24]. Wang et al found no correlation between plasma manganese and bone mineral density in an epidemiological study of 91 elderly men over 50 years old in Beijing [25]. However, in a cross-sectional study of 304 retired workers with occupational manganese exposure, aiming to investigate the relationship between long-term occupational manganese exposure and bone quality, female participants in the highest manganese exposure group were significantly lower than the female control group in terms of stiffness index (SI) and T-score levels. This suggests that women with occupational manganese exposure may have a higher risk of osteoporosis [14]. Different sample sizes, inclusion and exclusion criteria, duration of follow-up, and different levels of manganese exposure may lead to different results between studies. These results indicate that the relationship between blood manganese and bone mineral density is still uncertain, and further basic studies are needed to explore the relationship between manganese and bone metabolism.

Our study shows that blood manganese levels are associated with bone mineral density of femur and total body, and high blood manganese levels may affect bone metabolism. Bone is one of the body’s main organs for long-term storage of manganese, and according to the International Commission on Radiation Protection (ICRP), about 40% of body manganese accumulates in bones [17]. Evidence from animal study suggested that Mn accumulated in rat bones with an average elimination half-life of 143 days, equivalent to approximately 8.5 years in human bones [21]. Thus, bone may also be an ideal organ exploring toxicity which caused by long-term manganese exposure [14]. It has been previously reported that excessive manganese may be neurotoxic to humans, affecting specific areas of the central nervous system and producing irreversible neurological symptoms [26]. Occupational exposure concentrations of airborne manganese in the range of 0.1–1.27 mg/m^3^ indicate blood levels of 10.3–12.5 ug/ L [27]. In patients with clinical signs and symptoms of manganese poisoning, blood manganese concentration varies between 4 and 40 ug/L [21, 28–30]. Studies have shown that dietary manganese deficiency and excessive manganese exposure can increase the production of reactive oxygen species (ROS), and abnormal accumulation of excessive ROS will exacerbate oxidative stress and inflammation, which is crucial in the pathogenesis of metabolic diseases(31). Recent studies [31, 32] have found that during osteoblast differentiation, mitochondrial biogenesis, mitochondrial function (especially the activity of complex I in mitochondrial ECT) and ATP content are significantly increased, while excessive manganese accumulation in mitochondria inhibits mitochondrial complex I and II respiration and induces permeability transition [33–36]. It may disrupt mitochondrial homeostasis and lead to mitochondrial dysfunction, ultimately adversely affecting BMD. In addition, proposed in recent years, mitophagy has been reported that it improves tissue homeostasis via recovering energy by limiting the energy demands of ineffective organelles, reducing intracellular ROS produced by impaired mitochondria, and generating ATP during degradation under physiological or pathological conditions, while the disorder of mitophagy damages cells energy metabolism and physiological function, and breaks mitochondrial homeostasis [31, 37]. Thus, we speculate that excess manganese may also adversely affect bone metabolism through the mitophagy pathway, and the mechanism of this still needs further studied.

Significantly higher blood Mn levels have been revealed in women of US residents, Canadians, Koreans, Chinese general population, and Italians [14, 38–42]. Mining and iron as well as steel production are considered as occupational sources of manganese exposure. Humans are more likely to be exposed to manganese through diet. Nuts, grain products, chocolate, crustaceans, legumes, mollusks, fresh fruits and tea are considered as rich sources of manganese [43]. In the United States, the recommended daily intake is 1.8 mg/day for women and 2.3 mg/day for men [44]. Excessive intake of manganese has been reported to be associated with cognitive impairment in human [45, 46]. According to the National Institutes of Health, consuming more than 11 milligrams a day may have bad effects and the typical non-vegetarian Western diets provide 3–7 mg of manganese per day [47]. Previous studies have suggested that routine supplementation of manganese and other metals is generally unnecessary and such excessive supplementation may be harmful [44]. In the current study, we reported a negative association between blood manganese in adults selected from NHANES in 2013–2014 and 2017–2018 and femur and total body BMD/BMC. As the results showed, manganese intake may remain as an important public health issue. Some multivitamins typically contain 2–4 mg of additional manganese, and those with long-term intake, especially older adults, need to pay extra attention to their long-term risks and benefits. While in Europe, the European Food Safety Agency (EFSA), considering lacking of the sufficient and definitive data concerning humans, has neither established any tolerable upper intake Level (UL), nor an average requirement (Average Requirement, AR) and a reference intake for the population (population reference intake, PRI) for Mn [48, 49]. The results of our study may provide some references for the establishment of AR, PRI and UL of Mn. In addition, regular dual-energy radiographs of the femur or total body are recommended for people exposed to manganese, including those living near factories where manganese is utilized in production, especially women, to be aware of the risk of osteoporosis.

This study has several strengths for exploring the relationship between blood manganese status and BMD/BMC in different populations. (i) Based on data from the NHANES database, this study has a large sample size and sufficient clinical information. (ii) After adequately controlling for confounding factors, this study estimated the difference in the association between blood manganese status and BMD/BMC in diverse populations by stratifying age, sex and race. (iii) This is the first study that found decreased femoral neck BMC and BMD in female participants aged 50–70 had higher blood manganese levels, which suggests that blood manganese might have potential value in predicting osteopenia, osteoporosis and fragility fractures.

In addition, this study still has some limitations. (i) As a cross-sectional study, the causal relationship between Mn and BMD/BMC could be bidirectional. Nevertheless, great attention should be paid to the influence of blood manganese level in daily diet and life. More clinical trial and basic researches about the mechanism should be performed in the further. (ii) The total body BMD and BMC in the NHANES database only included data from samples aged 8–59 years (we excluded data under 18 years old), while the BMD and BMC of femur and spine only included data over 40 years old, which could not properly represent the American population over 18 years old. (iii) The study only enrolled the participants in the United States, so the results may not accurately apply to other countries or regions such as Asia and Africa. (iv) Residual or unmeasured confounders are possible despite adjustment for potential known confounders.

## Conclusion

This nationwide cross-sectional study has showed a negative association between blood manganese levels and femur as well as total body BMD/BMC, especially femoral neck BMD in women aged 50–70 years. People exposed to manganese should be aware of the increased risk of osteopenia or osteoporosis. Besides, due to the lack of available data, there are no definite values for the tolerable upper intake level (UL), average requirement (AR) and population reference intake (PRI) of manganese. The results of our study may provide some references for the establishment of AR, PRI and UL of Mn.

## Supporting information

S1 Table Related demographic characteristics of participants included in NHANES from 2013 to 2014 and 2017 to 2018, based on blood manganese quartiles. Means with standard deviation (SD) were used for continuous characteristic variables and categorical variables were expressed as frequencies. Differences in categorical variables between exposed groups were analyzed by Pearson’s chi-square tests. One-way ANOVA was used to analyze the differences of continuous variables between groups.
(DOCX)

S2 Table Blood manganese and area, BMC, BMD based on blood manganese quartiles. Data are expressed as means (standard deviation, SD). BMC, bone mineral content, BMD, bone mineral density, Area (cm^2^), BMC (g), BMD (g/cm^2^).
(DOCX)

## Abbreviations

AR: average requirement
BMC: bone mineral content
BMD: bone mineral density
DEXA: Dual Energy X-ray Absorptiometry
EFSA: European Food Safety Agency
ICRP: International Commission on Radiation Protection
NHANES: National Health and Nutrition Examination Survey
PRI: population reference intake
QALYs: quality-adjusted life years
ROS: reactive oxygen species
SI: stiffness index
UL: upper intake level
