# Supplementary material for: Relationship between blood manganese and bone mineral density and bone mineral content in adults: A population-based cross-sectional study
Source: PLoS One. 2022 Oct 21;17(10):e0276551. doi: 10.1371/journal.pone.0276551 (PMC9586363; doi:10.1371/journal.pone.0276551)
Supplement: S1 Table — Means with standard deviation (SD) were used for continuous characteristic variables and categorical variables were expressed as frequencies. Differences in categorical variables between exposed groups were analyzed by Pearson’s chi-square tests. One-way ANOVA was used to analyze the differences of continuous variables between groups. (DOCX) [file pone.0276551.s001.docx]

| **S1 Table Related demographic characteristics of participants Included in NHANES from 2013 to 2014 and 2017 to 2018** | | | | | | | | | | | | | | | | |
| --- | --- | --- | --- | --- | --- | --- | --- | --- | --- | --- | --- | --- | --- | --- | --- | --- |
| **Characteristic** | | **femur** | | | | | **spine** | | | | | **total body** | | | | |
|  |  | **low  manganese** | **normal  manganese** | **high  manganese** | **total** | **P value** | **low  manganese** | **normal  manganese** | **high  manganese** | **total** | **P value** | **low  manganese** | **normal  manganese** | **high  manganese** | **total** | **P value** |
| **Gender** | male | 61 | 1828 | 25 | 1914 | 0.0036 | 31 | 998 | 15 | 1044 | 0.0894 | 37 | 1774 | 23 | 1834 | <0.001 |
|  | female | 43 | 1746 | 49 | 1838 |  | 30 | 1189 | 35 | 1254 |  | 23 | 1709 | 116 | 1848 |  |
| **Age (years)** | | 65.3±10.3 | 60.3±10.7 | 58.8±11.1 | 62.1±10.7 | 0.0001 | 62.0±10.3 | 59.8±10.8 | 55.0±10.7 | 59.6±10.8 | 0.001 | 39.2±12.5 | 38.0±12.4 | 36.1±12.2 | 37.7+12.4 | 0.2452 |
| **Race** | Mexican American | 5 | 441 | 9 | 455 | <0.0001 | 3 | 306 | 7 | 316 | <0.001 | 3 | 564 | 24 | 591 | <0.001 |
|  | Other Hispanic | 7 | 361 | 9 | 377 |  | 5 | 235 | 8 | 248 |  | 7 | 355 | 12 | 374 |  |
|  | Non-Hispanic White | 37 | 1446 | 22 | 1505 |  | 20 | 781 | 15 | 816 |  | 22 | 1224 | 32 | 1278 |  |
|  | Non-Hispanic Black | 50 | 749 | 6 | 805 |  | 29 | 470 | 2 | 501 |  | 23 | 610 | 11 | 644 |  |
|  | Other Race - Including Multi-Racial | 5 | 577 | 28 | 610 |  | 4 | 395 | 18 | 417 |  | 5 | 730 | 60 | 795 |  |
| **Weight(kg)** | | 81.7±20.4 | 69.7±18.5 | 62.1±18.6 | 79.9±18.5 | 0.0323 | 79.5±20.6 | 67.6±17.3 | 59.1±21.5 | 79.2±18.7 | 0.1352 | 79.4±18.6 | 79.1±20.4 | 75.0±21.0 | 79.3±20.4 | 0.0264 |
| **Standing Height(cm)** | | 167.9±9.5 | 163.0±8.3 | 158.1±9.6 | 166.0±8.8 | 0.0278 | 166.6±10.1 | 162.0±8.4 | 159.2±8.9 | 165.1±8.6 | 0.0018 | 169.3±9.6 | 167.8±9.4 | 162.5±8.0 | 167.2±9.4 | <0.001 |
| **BMI (kg/m2)** | | 28.8±6.1 | 26.1±6.3 | 24.9±6.4 | 28.9±6.3 | 0.1281 | 28.4±6.1 | 25.7±6.4 | 23.3±7.0 | 28.9±6.6 | 0.3683 | 27.7±6.0 | 28.0±6.6 | 28.4±7.4 | 28.3±6.6 | 0.7875 |
| **Drinking alcohol** | YES | 18 | 526 | 10 | 554 | 0.8151 | 8 | 287 | 8 | 303 | 0.6733 | 15 | 376 | 12 | 403 | 0.0073 |
|  | NO | 71 | 2449 | 49 | 2569 |  | 39 | 1483 | 29 | 1551 |  | 39 | 2482 | 84 | 2605 |  |
|  | **N/A** | 15 | 599 | 15 | 629 |  | 14 | 417 | 13 | 444 |  | 6 | 625 | 43 | 674 |  |
| **Hypertension** | YES | 63 | 1794 | 35 | 1892 | 0.0999 | 37 | 1022 | 23 | 1082 | 0.1001 | 23 | 731 | 25 | 779 | 0.0033 |
|  | NO | 41 | 1775 | 39 | 1855 |  | 24 | 1162 | 27 | 1213 |  | 37 | 2749 | 113 | 2899 |  |
|  | **N/A** | 0 | 5 | 0 | 5 |  | 0 | 3 | 0 | 3 |  | 0 | 3 | 1 | 4 |  |
| **Hypercholesterolemia** | YES | 49 | 1758 | 30 | 1837 | 0.3139 | 30 | 1013 | 21 | 1064 | 0.8012 | 8 | 762 | 23 | 793 | 0.1002 |
|  | NO | 55 | 1783 | 43 | 1881 |  | 31 | 1153 | 28 | 1212 |  | 52 | 2702 | 114 | 2868 |  |
|  | **N/A** | 0 | 33 | 1 | 34 |  | 0 | 21 | 1 | 22 |  | 0 | 14 | 2 | 16 |  |
| **Diabetes** | YES | 28 | 701 | 16 | 745 | 0.1990 | 12 | 417 | 13 | 442 | 0.5191 | 2 | 216 | 7 | 225 | 0.5882 |
|  | NO | 73 | 2721 | 56 | 2850 |  | 48 | 1678 | 36 | 1762 |  | 56 | 3193 | 127 | 3376 |  |
|  | **N/A** | 3 | 152 | 2 | 157 |  | 1 | 92 | 1 | 94 |  | 2 | 74 | 5 | 81 |  |
| **Difficulty walking or climbing stairs** | YES | 29 | 631 | 14 | 674 | 0.0273 | 16 | 372 | 6 | 394 | 0.1052 | 4 | 166 | 7 | 177 | 0.7862 |
|  | NO | 75 | 2941 | 60 | 3076 |  | 45 | 1815 | 44 | 1904 |  | 56 | 3316 | 132 | 3504 |  |
|  | **N/A** | 0 | 2 | 0 | 2 |  | 0 | 0 | 0 | 0 |  | 0 | 1 | 0 | 1 |  |
| **Renal insufficiency** | YES | 13 | 159 | 5 | 177 | 0.0004 | 8 | 81 | 1 | 90 | 0.0006 | 2 | 57 | 1 | 60 | 0.4414 |
|  | NO | 90 | 3408 | 69 | 3567 |  | 52 | 2101 | 49 | 2202 |  | 55 | 3141 | 122 | 3318 |  |
|  | **N/A** | 1 | 7 | 0 | 8 |  | 1 | 5 | 0 | 6 |  | 3 | 285 | 16 | 304 |  |
| **Asthma** | YES | 17 | 490 | 6 | 513 | 0.2758 | 8 | 302 | 3 | 313 | 0.2789 | 9 | 551 | 17 | 577 | 0.5331 |
|  | NO | 87 | 3082 | 68 | 3237 |  | 53 | 1883 | 47 | 1983 |  | 51 | 2930 | 121 | 3102 |  |
|  | **N/A** | 0 | 2 | 0 | 2 |  | 0 | 2 | 0 | 2 |  | 0 | 2 | 1 | 3 |  |
| **Arthritis** | YES | 48 | 1436 | 16 | 1500 | 0.0023 | 28 | 803 | 11 | 842 | 0.0320 | 10 | 426 | 13 | 449 | 0.4300 |
|  | NO | 56 | 2127 | 58 | 2241 |  | 33 | 1379 | 39 | 1451 |  | 47 | 2770 | 110 | 2927 |  |
|  | **N/A** | 0 | 11 | 0 | 11 |  | 0 | 5 | 0 | 5 |  | 3 | 287 | 16 | 306 |  |
| **Congestive heart failure** | YES | 5 | 144 | 4 | 153 | 0.7594 | 2 | 79 | 2 | 83 | 0.9854 | 1 | 20 | 0 | 21 | 0.3764 |
|  | NO | 97 | 3418 | 69 | 3584 |  | 57 | 2099 | 48 | 2204 |  | 56 | 3179 | 123 | 3358 |  |
|  | **N/A** | 2 | 12 | 1 | 15 |  | 2 | 9 | 0 | 11 |  | 3 | 284 | 16 | 303 |  |
| **Coronary heart disease** | YES | 6 | 239 | 6 | 251 | 0.8214 | 3 | 112 | 6 | 121 | 0.1003 | 0 | 29 | 1 | 30 | 0.7666 |
|  | NO | 97 | 3321 | 67 | 3485 |  | 57 | 2066 | 44 | 2167 |  | 57 | 3168 | 122 | 3347 |  |
|  | **N/A** | 1 | 14 | 1 | 16 |  | 1 | 9 | 0 | 10 |  | 3 | 286 | 16 | 305 |  |
| **Angina pectoris** | YES | 4 | 239 | 3 | 246 | 0.3438 | 3 | 68 | 2 | 73 | 0.6976 | 1 | 31 | 1 | 33 | 0.8221 |
|  | NO | 100 | 3321 | 71 | 3492 |  | 58 | 2105 | 48 | 2211 |  | 56 | 3165 | 122 | 3343 |  |
|  | **N/A** | 0 | 14 | 0 | 14 |  | 0 | 14 | 0 | 14 |  | 3 | 287 | 16 | 306 |  |
| **Stroke** | YES | 10 | 196 | 2 | 208 | 0.1086 | 3 | 110 | 2 | 115 | 0.9455 | 2 | 47 | 0 | 49 | 0.1730 |
|  | NO | 94 | 3371 | 72 | 3537 |  | 58 | 2073 | 48 | 2179 |  | 55 | 3152 | 123 | 3330 |  |
|  | **N/A** | 0 | 6 | 0 | 6 |  | 0 | 4 | 0 | 4 |  | 3 | 284 | 16 | 303 |  |
| **Thyroid problems** | YES | 13 | 536 | 13 | 562 | 0.6179 | 6 | 317 | 11 | 334 | 0.1699 | 5 | 221 | 12 | 238 | 0.4228 |
|  | NO | 91 | 3029 | 60 | 3180 |  | 55 | 1865 | 38 | 1958 |  | 52 | 2975 | 111 | 3138 |  |
|  | **N/A** | 0 | 9 | 1 | 10 |  | 0 | 5 | 1 | 6 |  | 3 | 287 | 16 | 306 |  |
| **COPD** | YES | 10 | 222 | 6 | 238 | 0.3104 | 5 | 111 | 5 | 121 | 0.1800 | 2 | 42 | 0 | 44 | 0.1508 |
|  | NO | 94 | 3344 | 68 | 3506 |  | 56 | 2071 | 45 | 2172 |  | 55 | 3155 | 123 | 3333 |  |
|  | **N/A** | 0 | 8 | 0 | 8 |  | 0 | 5 | 0 | 5 |  | 3 | 286 | 16 | 305 |  |
| **Smoking** | YES | 49 | 1645 | 31 | 1725 | 0.7577 | 24 | 929 | 18 | 971 | 0.5891 | 27 | 1240 | 33 | 1300 | 0.0047 |
|  | NO | 55 | 1929 | 43 | 2027 |  | 37 | 1258 | 32 | 1327 |  | 33 | 2243 | 106 | 2382 |  |
|  | **N/A** | 0 | 0 | 0 | 0 |  | 0 | 0 | 0 | 0 |  | 0 | 0 | 0 | 0 |  |
| **Household smokers** | YES | 30 | 862 | 20 | 912 | 0.4555 | 15 | 549 | 12 | 576 | 0.9471 | 23 | 1013 | 36 | 1072 | 0.1842 |
|  | NO | 70 | 2603 | 53 | 2726 |  | 45 | 1574 | 38 | 1657 |  | 36 | 2374 | 103 | 2513 |  |
|  | **N/A** | 4 | 109 | 1 | 114 |  | 1 | 64 | 0 | 65 |  | 1 | 96 | 0 | 97 |  |
| **Osteoporosis** | YES | 12 | 375 | 8 | 395 | 0.9463 | 10 | 223 | 3 | 236 | 0.1785 | 2 | 44 | 0 | 46 | 0.1907 |
|  | NO | 92 | 3182 | 66 | 3340 |  | 51 | 1951 | 47 | 2049 |  | 19 | 1131 | 35 | 1185 |  |
|  | **N/A** | 0 | 17 | 0 | 17 |  | 0 | 13 | 0 | 13 |  | 39 | 2308 | 104 | 2451 |  |

Means with standard deviation (SD) were used for continuous characteristic variables and categorical variables were expressed as frequencies. Differences in categorical variables between exposed groups were analyzed by Pearson’s chi-square tests. One-way ANOVA was used to analyze the differences of continuous variables between groups.
