# Supplementary material for: Relationship between blood manganese and bone mineral density and bone mineral content in adults: A population-based cross-sectional study
Source: PLoS One. 2022 Oct 21;17(10):e0276551. doi: 10.1371/journal.pone.0276551 (PMC9586363; doi:10.1371/journal.pone.0276551)
Supplement: S2 Table — Data are expressed as means (standard deviation, SD). BMC, bone mineral content, BMD, bone mineral density, Area (cm2), BMC (g), BMD (g/cm2). (DOCX) [file pone.0276551.s002.docx]

| **S2 Table Blood manganese and area, BMC, BMD based on blood manganese quartiles** | | | | | | | | | | | | |
| --- | --- | --- | --- | --- | --- | --- | --- | --- | --- | --- | --- | --- |
|  | Q1 | Q2 | Q3 | Q4 | P for trend |  | Q1 | Q2 | Q3 | Q4 | P for trend |  |
| Head Area | 227.3±17.2 | 225.9±17.5 | 224.9±18 | 224.2±17.8 | 0.0007 | Total femur BMD | 0.9±0.2 | 0.9±0.2 | 0.9±0.2 | 0.9±0.2 | 0.0043 |  |
| Head BMC | 511.1±89.4 | 499±90.6 | 501±88.4 | 502.3±90.9 | 0.0202 | Total femur BMC | 37.5±9.7 | 36.3±9.8 | 35.1±9.8 | 33.2±9.2 | <0.001 |  |
| Head BMD | 2.2±0.3 | 2.2±0.4 | 2.2±0.3 | 2.2±0.4 | 0.0799 | Total femur area | 39.6±6.3 | 38.3±6.2 | 37.3±6.2 | 35.8±6.1 | <0.0001 |  |
| Left Arm Area | 237.5±36.4 | 231.6±36.2 | 226.1±35.9 | 212.8±32.6 | <0.0001 | Femoral neck BMD | 0.8±0.1 | 0.8±0.1 | 0.8±0.1 | 0.8±0.1 | 0.0336 |  |
| Left Arm BMC | 190.7±46.8 | 182.9±46.5 | 175.1±45.4 | 158.3±39.7 | <0.0001 | Femoral neck BMC | 4.2±0.9 | 4.1±0.9 | 4±1 | 3.8±0.9 | <0.001 |  |
| Left Arm BMD | 0.8±0.1 | 0.8±0.1 | 0.8±0.1 | 0.7±0.1 | <0.0001 | Femoral neck area | 5.4±0.6 | 5.3±0.6 | 5.2±0.6 | 5.1±0.6 | <0.0001 |  |
| Left Leg Area | 379.7±48 | 371.4±49.4 | 364.7±48.2 | 345.3±44.4 | <0.0001 | Trochanter BMD | 0.7±0.1 | 0.7±0.1 | 0.7±0.1 | 0.7±0.1 | 0.1001 |  |
| Left Leg BMC | 458.4±99.9 | 443.6±101.4 | 428.7±94.8 | 394.4±85.8 | <0.0001 | Trochanter BMC | 9±2.6 | 8.7±2.5 | 8.4±2.5 | 8±2.5 | <0.0001 |  |
| Left Leg BMD | 1.2±0.1 | 1.2±0.1 | 1.2±0.1 | 1.1±0.1 | <0.0001 | Trochanter area | 12.7±2.3 | 12.2±2.1 | 11.9±2.2 | 11.4±2.3 | <0.0001 |  |
| Right Arm Area | 242.1±35 | 235.8±35.2 | 231±34.8 | 217.8±32.1 | <0.0001 | Intertrochanter BMD | 1.1±0.2 | 1.1±0.2 | 1.1±0.2 | 1.1±0.2 | 0.0043 |  |
| Right Arm BMC | 199.8±47.5 | 191.6±47.9 | 183.6±46.4 | 165.9±40.4 | <0.0001 | Intertrochanter BMC | 24.4±6.8 | 23.5±6.8 | 22.6±6.8 | 21.3±6.4 | <0.0001 |  |
| Right Arm BMD | 0.8±0.1 | 0.8±0.1 | 0.8±0.1 | 0.8±0.1 | <0.0001 | Intertrochanter area | 21.6±4.1 | 20.8±4 | 20.1±4 | 19.3±4 | <0.0001 |  |
| Right Leg Area | 379.4±48.2 | 371.3±48.8 | 364.6±48.3 | 345.6±44.2 | <0.0001 | Wards triangle BMD | 0.6±0.2 | 0.6±0.2 | 0.6±0.2 | 0.6±0.2 | 0.2455 |  |
| Right Leg BMC | 461.6±103.1 | 446.7±101.9 | 432.2±96.4 | 397.6±85.9 | <0.0001 | Wards triangle BMC | 0.7±0.2 | 0.7±0.2 | 0.7±0.2 | 0.7±0.2 | 0.2371 |  |
| Right Leg BMD | 1.2±0.1 | 1.2±0.1 | 1.2±0.1 | 1.1±0.1 | <0.0001 | Wards triangle area | 1.2±0.1 | 1.2±0.1 | 1.2±0.1 | 1.2±0.1 | 0.2714 |  |
| Left Ribs Area | 130.3±20 | 128.8±20.8 | 127.7±20.3 | 124.2±19.6 | <0.0001 | Total spine BMD | 1±0.2 | 1±0.2 | 1±0.2 | 1±0.2 | 0.003 |  |
| Left Ribs BMC | 86.2±19.6 | 84.1±19.6 | 81.9±19.1 | 77.7±17.6 | <0.0001 | Total spine BMC | 64±16.5 | 61.5±15.7 | 60.2±15.6 | 58±14.3 | <0.0001 |  |
| Left Ribs BMD | 0.7±0.1 | 0.6±0.1 | 0.6±0.1 | 0.6±0.1 | <0.0001 | Total spine area | 62.5±9 | 60.4±8.3 | 59.6±8.6 | 58.8±8 | <0.0001 |  |
| Thoracic Spine Area | 144.8±20.6 | 146.6±20.5 | 146±19.6 | 141.2±18.4 | <0.0001 | L1 BMD | 1±0.2 | 1±0.2 | 0.9±0.2 | 0.9±0.2 | 0.0212 |  |
| Thoracic Spine BMC | 121.7±27.4 | 122.6±26.9 | 121.4±27 | 116±24.6 | <0.0001 | L1 BMC | 13.6±3.8 | 13.1±3.5 | 12.8±3.4 | 12.3±3.3 | <0.0001 |  |
| Thoracic Spine BMD | 0.8±0.1 | 0.8±0.1 | 0.8±0.1 | 0.8±0.1 | 0.0014 | L1 area | 14.1±2.3 | 13.6±1.9 | 13.4±1.9 | 13.2±1.8 | <0.0001 |  |
| Lumbar Spine Area | 54.8±8.3 | 54.5±8.5 | 53.7±8.3 | 51.6±7.5 | <0.0001 | L2 BMD | 1±0.2 | 1±0.2 | 1±0.2 | 1±0.2 | 0.0094 |  |
| Lumbar Spine BMC | 57.8±13.9 | 56.7±13.6 | 55.6±12.9 | 52.8±11.8 | <0.0001 | L2 BMC | 15.2±4 | 14.6±4 | 14.4±3.8 | 13.8±3.6 | <0.0001 |  |
| Lumbar Spine BMD | 1±0.2 | 1±0.1 | 1±0.1 | 1±0.1 | <0.0001 | L2 area | 14.9±2.2 | 14.4±2.1 | 14.3±2 | 14±2 | <0.0001 |  |
| Pelvis Area | 208.1±40.1 | 202.4±38.7 | 197.5±38.1 | 186.8±34 | <0.0001 | L3 BMD | 1±0.2 | 1±0.2 | 1±0.2 | 1±0.2 | 0.0042 |  |
| Pelvis BMC | 265±73.8 | 255.6±70.2 | 249.3±68.9 | 230.4±59.9 | <0.0001 | L3 BMC | 17±4.4 | 16.5±4.2 | 16±4.1 | 15.5±3.8 | <0.0001 |  |
| Pelvis BMD | 1.3±0.2 | 1.3±0.2 | 1.2±0.2 | 1.2±0.2 | <0.0001 | L3 area | 16.2±2.4 | 15.7±2.1 | 15.4±2.2 | 15.2±2 | <0.0001 |  |
| Trunk Bone area | 672.7±81.2 | 666.5±81.3 | 659.8±81.3 | 634.9±75.7 | <0.0001 | L4 BMD | 1±0.2 | 1±0.2 | 1±0.2 | 1±0.2 | 0.002 |  |
| Trunk BMC | 617.9±132.9 | 605±128.6 | 593.3±127.1 | 557.4±113 | <0.0001 | L4 BMC | 18.6±4.9 | 17.6±4.7 | 17.5±4.6 | 16.8±4.6 | <0.0001 |  |
| Trunk Bone BMD | 0.9±0.1 | 0.9±0.1 | 0.9±0.1 | 0.9±0.1 | <0.0001 | L4 area | 17.7±2.6 | 17±2.4 | 16.9±2.5 | 16.6±2.4 | <0.0001 |  |
| Subtotal Area | 1911.3±229 | 1876.5±233.9 | 1846.1±230.3 | 1756.4±212.7 | <0.0001 | Total Area | 2138.7±237.4 | 2102.4±242.5 | 2071±238.6 | 1980.5±219.3 | <0.0001 |  |
| Subtotal BMC | 1928.5±405.7 | 1869.8±407.1 | 1812.9±389.9 | 1673.6±346.9 | <0.0001 | Total BMC | 2439.6±447.5 | 2368.8±449.5 | 2313.9±428.8 | 2175.9±385 | <0.0001 |  |
| Subtotal BMD | 1±0.1 | 1±0.1 | 1±0.1 | 0.9±0.1 | <0.0001 | Total BMD | 1.1±0.1 | 1.1±0.1 | 1.1±0.1 | 1.1±0.1 | <0.0001 |  |

Data are expressed as means (standard deviation, SD). BMC, bone mineral content, BMD, bone mineral density, Area (cm^2^), BMC (g), BMD (g/cm^2^)
